# Mauriac syndrome and early cataract diabetic

**DOI:** 10.1186/1687-9856-2013-S1-P1

**Published:** 2013-10-03

**Authors:** Eka Agustia Rini, Kemala Sayuti, Amirah zatil Izzah, Elfitrimelly

**Affiliations:** 1 Pediatric Endocrinology, Medical Faculty of Andalas University, Padang City, West Sumatra, Indonesia / dr.M.Jamil Hospital Padang West Sumatra, Indonesia; 2 Pediatric Ophthalmology, Medical Faculty of Andalas University, Padang City, West Sumatra, Indonesia / dr.M.Jamil Hospital Padang West Sumatra, Indonesia

## Introduction

Mauriac syndrome (MS) is characterized by development of dwarfism, obesity and hepatomegaly in patients with insulin dependent diabetes mellitus (IDDM). Growth retardation and hepatomegaly in IDDM should alert physicians over insufficient management of DM.

## Aim

To alert medical community of importance in preventing and managing diabetic complication.

## Result

A 5 year and 10 month old boy, was admitted with protuberant abdomen since one year ago. He had been recognized as type 1 DM and treated by subcutaneous insulin 4-5 U/day twice a day until admission.His abdomen became larger, wasted arms and legs so he couldn’t support his body, and just laid down for 3 months. He had retarded growth and development. Oedema face, chest was normal, distended abdomen with hepatomegaly and ascites. Extremities were disuse atrophy and pitting oedema. Laboratory investigation were; blood glucose 1074 mg/dl, metabolic acidosis, elevated of liver enzyme, hypoalbuminemia, hyperlipidemia and positive ketone bodies. HbA1c, C-peptide and IAA values were > 18,5%, 1,2 ng/ml and 3,9 u/ml respectively. Bone age was retarded boy. At 10^th^ day, oedema and ascites had disappeared. Six months later, he diagnosed with cataract dextra et sinistra by ophtalmologist and undergone cataract surgery. Growth velocity was 18 cm/ year (catch up growth); liver was ¼-1/4. HbA1c value was 9,9%. Insulin dose was 1 U/kg/day.

## Conclusion

Mauriac syndrome can resolve with appropriate insulin dose, good glycemic control, and improved growth velocity. Early cataract occurs in poor glycemic control.

